# PReS-FINAL-2297: Atmospheric pollution: influence on disease activity in childhood-onset systemic lupus erythematosus patients

**DOI:** 10.1186/1546-0096-11-S2-P287

**Published:** 2013-12-05

**Authors:** LM Campos, EGC Fernandes, CA Silva, AA Braga, AM Sallum, SCL Farhat

**Affiliations:** 1 Paediatric Rheumatology Unit, Children's Institute, University of São Paulo, São Paulo, Brazil; 2 Laboratory of Experimental Air Pollution, University of São Paulo, São Paulo, Brazil

## Introduction

Environmental factors, such as atmospheric pollution, may trigger the inflammation in adult systemic lupus erythematosus (SLE) patients. However, the role of atmospheric pollution and disease activity in childhood-onset systemic lupus erythematosus (C-SLE) population was not reported at this moment.

## Objectives

To investigate the association between changes in daily concentrations of air pollutants in São Paulo metropolitan region and disease activity in C-SLE patients.

## Methods

This was a longitudinal panel study including 410 consecutive medical visits in 22 C-SLE patients (ACR criteria). They were followed at the Pediatric Rheumatology Unit, Children's Institute, Faculdade de Medicina da Universidade de Sao Paulo, Brazil, between 2005 and 2010. Disease activity was evaluated according to Systemic Lupus Erythematosus Disease Activity Index 2000 (SLEDAI-2K), and the patients were divided arbitrarily in two groups: with disease activity (SLEDAI>8) and without disease activity (SLEDAI<8). The São Paulo State Environmental Agency (CETESB) provided daily concentrations of inhaled particulate matter (PM_10_), sulfur dioxide (SO_2_), nitrogen dioxide (NO_2_), ozone (O_3_) and carbon monoxide (CO). Meteorological variables, such as the minimum temperature and relative humidity, were obtained from the Institute of Astronomy and Geophysics of the University of São Paulo. Generalized estimation equation (GEE) model were used for binomial distribution to assess the impact of these measurements in the SLEDAI 2K score, considering the fixed effects for repetitive measurements, and adjusted for erythrocyte sedimentation rate, C-reactive protein, prednisone and/or immunosuppressant use, presence of infection 20 days before the medical appointment, minimum temperature and relative humidity. The results were expressed in relative risk (RR) and confidence interval (CI) of 95%.

## Results

410 consecutive medical visits were evaluated in 22 C-SLE patients (20 girls), with a mean of 19 visits/patient (4-30). The mean current age at the time of evaluation was 15.3 years (10.8-19.0). The mean age at C-SLE diagnosis was 10.3 years (6-12) and the mean age at disease duration was 5.3 years (7 months-11 years). Interquartile range increases of PM_10 _(25.2 μg/m^3^), CO (0.8 μg/m^3^), and NO_2 _(102 μg/m^3^) were associated with increases of 1.74 (CI of 95% 1.28-2.39), 1.37 (CI 95% 1.12-1.67) and 1.11 (CI 95% 1.02-1.21) in the risk of SLEDA-2K score score>8), respectively, 13 days after the exposure to these pollutants. The four days PM_10 _cumulative effect (from lag13 to lag16) increased the risk of outburst of SLE in 65% (CI 95% 1.06- 2.75). In contrast, ozone and SO_2 _did not show a significant effect on the SLEDAI-2K score.

## Conclusion

Variations in air pollution may influence disease activity in C-SLE patients. Therefore, oxidative stress may be an important trigger of inflammation in this systemic autoimmune disease.

## Disclosure of interest

None declared.

